# Expertise in Clinical Psychology. The Effects of University Training and Practical Experience on Expertise in Clinical Psychology

**DOI:** 10.3389/fpsyg.2013.00141

**Published:** 2013-03-27

**Authors:** Sabine Vollmer, Hans Spada, Franz Caspar, Salome Burri

**Affiliations:** ^1^Department of Psychology, University of FreiburgFreiburg, Germany; ^2^Department of Clinical Psychology and Psychotherapy, University of BernBern, Switzerland

**Keywords:** expertise, experience, clinical knowledge, clinical competencies, behavior therapy

## Abstract

How do university training and subsequent practical experience affect expertise in clinical psychology? To answer this question we developed methods to assess psychological knowledge and the competence to diagnose, construct case conceptualizations, and plan psychotherapeutic treatment: a knowledge test and short case studies in a first study, and a complex, dynamically evolving case study in the second study. In our cross-sectional studies, psychology students, trainees in a certified postgraduate psychotherapist curriculum, and behavior therapists with more than 10 years of experience were tested (100 in total: 20 each of novice, intermediate, and advanced university students, postgraduate trainees, and therapists). Clinical knowledge and competence increased up to the level of trainees but unexpectedly decreased at the level of experienced therapists. We discuss the results against the background of expertise research and the training of clinical psychologists (in Germany). Important factors for the continuing professional development of psychotherapists are proposed.

## Introduction

In our studies we examined in detail the degree to which expertise in clinical psychology and psychotherapy develops at different points in the course of education at university level, training at the postgraduate level, and subsequent practical experience. Based on psychotherapy research and findings of cognitive science on expertise in general we developed instruments for assessing knowledge in clinical psychology as well as psychotherapeutic competencies. In two cross-sectional studies, German university students at different stages of their studies, graduate therapist trainees, and experienced psychotherapists were assessed.

In the following section, we provide a brief overview of expertise research with a special focus on expertise in medicine as a relatively well explored neighboring field. Moreover, results from psychotherapy research on clinical expertise are summarized.

Research on the nature of expertise has attracted a great deal of attention since the 1960s until the present (for an overview, see Ericsson et al., 2006). There are some common features shared by experts as opposed to novices in most of the studies (VanLehn, 1989; Bédard and Chi, 1992; Feltovich et al., 2006): experts have acquired a vast and well-connected knowledge base. Confronted with a problem situation, they are able to recall more relevant items and are better able to identify pertinent information and disregard irrelevant information. Experts are able to perform domain specific tasks faster than novices and they solve domain specific problems more correctly than novices. These general results found in various domains provide a basis for studying expertise in new and more complex domains.

Psychotherapy is a professional domain for which certified training, partially at universities, has been developed in many countries to guarantee a professional standard. To examine expertise development in such a professional domain we have to relate formal training and practical experience to the development of expertise in order to obtain meaningful results. Like psychotherapy, medicine may also be characterized by the complexity of problems and its professional status and training. Thus, results of research on expertise development in the domain of medicine were of particular interest for planning our studies on expertise development in clinical psychology and psychotherapy. A broad and well-connected knowledge base (e.g., Elstein et al., 1978; Boshuizen and Schmidt, 1992; Van de Wiel et al., 2000) helps the expert medical practitioner to generate correct hypotheses faster (e.g., Patel and Groen, 1991) and diagnose more correctly, even under time pressure (Custers et al., 1996). In contrast to studies in other domains, medical experts do not recall more items after reading a written case. This is attributed to a process often called “knowledge encapsulation” (Boshuizen and Schmidt, 1992). In this process, higher-order concepts are developed under which lower-order concepts are subsumed. In routine work, experts verbalize only higher-order concepts. However, if asked to do so, or when problems arise, experts are able to reflect lower-order concepts and their connections. Recently, Marsh and Ahn (2012) also found knowledge encapsulation in the memory of experienced mental health clinicians.

Findings on expertise development in medicine may serve as a starting point for analyzing expertise in clinical psychology. However, three main differences between clinical psychologists and physicians have been described (Kingsbury, 1987): (1) medical students learn to view science as a body of facts, whereas students of psychology focus on scientific methods that help to test theories. (2) For physicians, there is a stronger association between particular diagnoses and specialized treatments. (3) Medical students usually start their studies with the goal of becoming a physician; clinical training is introduced relatively early. In contrast, the first years of the psychology curriculum at the university are oriented toward a scientific education focusing on methods and general concepts of psychology.

University education in Psychology and Psychotherapy in Germany can be characterized as: the content of the basic psychology curriculum at the university level is similar for nearly all German universities and resembles the core of the curricula of many universities worldwide. We describe the University of Freiburg’s psychology diploma curriculum (Diplom-Studiengang) as we recruited our sample from a population of students and postgraduates studying or having studied this curriculum^1^. In the first 2 years of their studies, students learn about basic principles of psychology and are encouraged to a critical and analytical mind-set. During their third year, students learn about the application of basics to different fields of psychological work, such as clinical psychology. They learn for instance about clinical disorder patterns and psychotherapeutic techniques. Students also improve their diagnostic competence, they learn for instance about clinical assessment methods and have their first courses on psychological interventions. In the fourth and fifth year, students can choose clinical psychology as one of their majoring subjects. They begin to solve realistic and complex problems and learn to interact with patients. They are also required to complete supervised internships. In the final phase of their studies, students write a thesis.

In Germany since 1999, psychologists intending to work as Psychological Psychotherapists are legally required to engage in a certified postgraduate training program of 3–5 years. These postgraduate education and training programs comprise theoretical and practical units as well as self experience or undergoing therapy. The theoretical units consist of psychotherapy-related lessons, e.g., cover various psychological disorders or elements of behavior therapy. In the practical units, trainees intern in a certified psychiatric ward as well as in a psychotherapeutic institution practicing to adapt their theoretical knowledge and competencies to individual cases. Once they have learned the basics they treat patients under supervision, mostly in outpatient settings. Psychologists who have finished this additional training are then legally allowed to work independently with patients. It should be noted that therapists who had completed their training before 1999 did not have to undergo such a full certified training.

But what is known already about development of expertise in clinical psychology and psychotherapy? Research on expertise development in clinical psychology and psychotherapy is still scarce (Betan and Binder, 2010). Currently, in the US it is argued that before being allowed to begin their clinical internship, students should prove that they possess a certain level of scientific knowledge as measured by the Examination for Professional Practice in Psychology (Stedman and Schoenfeld, 2011). Knowledge also underlies most of the competencies delineated by the APA Presidential Task Force on Evidence-Based Practice (APA, 2006). For instance, the competence of choosing adequate assessments, making meaningful diagnostic judgments, developing systematic case conceptualizations, and planning treatment can only be performed well if psychotherapists possess knowledge about psychopathology, diagnostic instruments, interventions, treatments, and treatment-patient-interactions.

Even though a strong and integrated knowledge base forms the basis of clinical expertise, knowledge also must be adapted and tailored to specific circumstances in order to be helpful in constructing systematic case conceptualizations for individual patients. Systematic case conceptualization has been considered a core competency in psychotherapy fostering accurate diagnoses, tailored treatment plans, and positive therapy outcomes (e.g., Eells et al., 2005; APA, 2006; Eells, 2007; Betan and Binder, 2010). Betan and Binder (2010) described case conceptualizations as “a working hypothesis about what causes, precipitates, and maintains a person’s psychological, interpersonal, and behavioral difficulties” (p. 143). While working on a systematic case conceptualization, the therapist develops a theory about what troubles the patient and what constraints and resources have to be taken into account. In accordance with Grawe’s general change mechanisms of psychotherapy (Grawe, 2004), we propose the following main components of an individual case conceptualization: therapy motivation, behavior in interpersonal relationships, resources, critical life events, and disorder-specific components. This comprehensive approach emphasizes potential starting points for therapeutic interventions and resources. It encompasses many components other authors have addressed in case formulations. Persons (2008), for instance, described elements and the process of case formulation for cognitive – behavioral therapy. She included elements like problem and disorder-specific behavior, cognitive-behavioral mechanisms, and cognitive-behavioral explanations (Persons, 2008). See Eells (2007) for an overview on other case conceptualizations.

Therapists with substantial expertise regarding case conceptualizations have been shown to outperform therapists with only a considerable amount of practical experience and novices in the quality of case conceptualizations as well as treatment plans (Eells et al., 2005).

Another relevant aspect of clinical expertise is the ability to develop valid diagnoses based on underlying knowledge and systematic case conceptualizations. Using case descriptions, Witteman and van den Bercken (2007) examined the quality of diagnoses developed by novices, intermediates, and experienced psychotherapists. They found that intermediates performed worse than novices and experienced psychotherapists. The experienced psychotherapists did not diagnose significantly more accurately than novices, which was a disappointing result. Similar negative effects were found regarding the diagnostic competence of experienced counselors (Witteman et al., 2012). The effects of years of experience on therapy outcome are unclear, effect sizes range from small negative effects to large positive effects (e.g., Beutler et al., 2004).

However, none of the above described studies compared psychotherapists with different levels of experience regarding the quality of diagnoses, case conceptualizations, and treatment planning.

## Research Questions

This study examines core components of expertise in the domain of clinical psychology and psychotherapy. We assessed clinical expertise with a multi-method approach comprising measurements of psychological knowledge and clinical competencies. The measurements varied in terms of complexity and in the way they captured the manifold demands of clinical practice. We studied the development of clinical expertise in detail in two cross-sectional studies. Our samples consisted of university students at different stages of studying psychology, graduate trainee therapists, and psychological behavior therapists with substantial clinical experience.

### Hypothesis 1

Effect of studying psychology on expertise in clinical psychology: we assumed that expertise development in clinical psychology and psychotherapy mirrors the way university training is organized. Accordingly, we expected that basic psychological knowledge would reach its maximum quite early in studying psychology (novice students) and knowledge of how to apply basic psychological knowledge to clinical psychology would reach its maximum in the third year of university studies (intermediate students), whereas clinical knowledge and clinical competencies would increase steadily with continuing formal education during the years of studying psychology (up to advanced students). Accordingly we assumed an increase of higher-order concepts in recalling relevant information of case studies comparable to the knowledge encapsulations found in the medical domain (Boshuizen and Schmidt, 1992). A detailed description of the instruments for assessing knowledge and clinical competencies is provided in the Section “Materials and Methods.”

### Hypothesis 2

Effect of postgraduate training on expertise in clinical psychology: we likewise assumed that postgraduate training would be reflected in expertise development, i.e., no further increase in basic psychological knowledge and application of basic psychological knowledge to clinical knowledge but a clear increase in clinical knowledge and competencies and additionally in the use of higher-order concepts.

### Hypothesis 3

Effect of practical clinical experience on expertise in clinical psychology: predictions for the effects of clinical experience in the years after studying psychology and clinical training were not as unequivocal. We assumed that if several years had elapsed since university training, even some decrease in basic knowledge of psychology might be the case. We expected the application of basic knowledge to clinical psychology to level off. But increased therapeutic experience should lead to deepened knowledge about clinical psychology and to enhanced clinical competencies. Accordingly the use of higher-order concepts should increase.

Study 1 quantitatively tested all these hypotheses. Study 2 supplemented the results of Study 1 with qualitative analyses of a smaller sample working on a very realistic and complex case study.

## Study 1

Based on expertise research, the results of psychotherapy research, and considering the features of formal psychology training, Study 1 quantitatively tested Hypotheses 1–3 with a multi-method approach examining the knowledge and competencies of individuals with different experience levels in clinical psychology and psychotherapy.

### Materials and methods

#### Participants and design

University students of psychology and psychologists comprising a total of five different levels of experience in clinical psychology participated in our study. Experience Levels 1–4 mirrored important steps in formal psychology training described above. Experience Level 5 consisted of experienced therapists who had been working as behavior therapists for at least 10 years. Altogether, 55 students (20 novice students: Level 1, 20 intermediate students: Level 2, and 15 advanced students with clinical focus: Level 3), 15 graduate trainee therapists (Level 4), and 15 experienced therapists (Level 5) participated in our study. Novice and intermediate students were on average 24 years old, advanced students 25 years, postgraduate trainee therapists 31 years, and the experienced therapists 49 years. As psychology is a popular subject for women in Germany, our sample was mainly female: 85% of the novice students were female, 80% of the intermediate students, 87% of the advanced students, 80% of the trainee therapist, and 73% of the experienced therapists.

All participants received a small financial compensation for their voluntary participation. Students and trainee psychotherapists were recruited during lectures and workshops. The psychotherapists were found *via* a special search engine for physicians and therapists^2^ and contacted by phone by the first author and research assistants. It should be noted that there may be a cohort effect, as the experienced therapists had not undergone a systematic postgraduate professional training comparable to that which therapists receive today.

#### Material and dependent variables

For an overview of the dependent variables please see Tables 1– 3 in the Section “Results.” These variables were measured using a computerized questionnaire with multiple-choice items, open-format questions, and short case studies. The complete questionnaire is available in German on^3^.

**Table 1:** **Means and standard deviations (in parentheses) for the knowledge test with multiple-choice items and open-format questions**.

Knowledge test	Novices	Intermediates	Advanced	Trainees	Therapists
**BASIC PSYCHOLOGICAL KNOWLEDGE**					
Multiple-choice percentage correct	77.0 (27.7)	73.0 (22.7)	69.3 (23.7)	68.0 (18.2)	53.3 (24.7)
Open-format: correct statements	3.75 (3.92)	2.05 (1.38)	2.13 (1.20)	2.53 (1.90)	1.90 (1.43)
Open-format: technical terms	2.70 (3.20)	1.20 (1.20)	0.60 (0.74)	1.47 (2.00)	1.07 (1.22)
**KNOWLEDGE OF HOW TO APPLY BASIC PSYCHOLOGICAL KNOWLEDGE TO CLINICAL PSYCHOLOGY**					
Multiple-choice percentage correct	56.6 (30.8)	85.0 (25.3)	84.4 (17.2)	84.4 (21.3)	80.0 (27.6)
**CLINICAL KNOWLEDGE**					
Multiple-choice percentage correct	41.3 (26.0)	51.3 (25.0)	60.0 (26.4)	83.3 (20.4)	68.3 (20.0)
Open-format: correct statements	1.10 (1.32)	2.93 (2.57)	3.63 (2.24)	5.92 (3.01)	2.40 (1.84)
Open-format: technical terms	0.10 (0.45)	1.45 (2.42)	2.27 (2.22)	4.07 (3.33)	1.00 (1.20)

**Table 2:** **Means and standard deviations (in parentheses) for Case Study 1**.

Case Study 1	Novices	Intermediates	Advanced	Trainees	Therapists
**RECALL**					
Correct statements	10.10 (2.94)	11.90 (5.03)	11.27 (5.00)	10.80 (4.20)	5.67 (3.09)
Higher-order concepts	1.30 (1.22)	1.60 (1.19)	2.27 (1.87)	3.53 (2.36)	2.13 (1.25)
**CLINICAL COMPETENCIES**					
Diagnosis correctness	0.51 (0.29)	0.75 (0.11)	0.76 (0.11)	0.80 (0.13)	0.68 (0.22)
Explanation correct statements	0.73 (0.45)	1.20 (0.90)	1.20 (0.64)	1.75 (0.91)	1.23 (0.76)

**Table 3:** **Means and standard deviations (in parentheses) for Case Study 2**.

Case Study 2	Novices	Intermediates	Advanced	Trainees	Therapists
**RECALL**					
Correct statem.	6.85 (2.89)	8.45 (3.28)	8.53 (4.49)	8.47 (3.31)	6.20 (4.49)
Higher-order concepts	1.50 (1.28)	1.25 (1.16)	2.27 (1.62)	2.53 (1.19)	1.87 (0.92)
**CLINICAL COMPETENCIES**					
Diagnosis correctness	0.36 (0.36)	0.59 (0.36)	0.61 (0.32)	0.68 (0.26)	0.69 (0.34)
Explanation correct statem.	0.13 (0.21)	0.79 (0.83)	0.77 (0.66)	1.12 (0.86)	0.73 (0.62)

##### Knowledge test

The questionnaire was comprised of multiple-choice questions and open-format questions on (a) basic psychological knowledge, (b) knowledge about how to apply basic psychological knowledge to clinical psychology, and (c) clinical knowledge. (a) Basic psychological knowledge was assessed by five multiple-choice questions and one open-format question (e.g., the open-format question asked participants to write down everything they knew about schedules of reinforcement). (b) Knowledge regarding the application of basics to clinical psychology, e.g., Mowrer’s two-factor theory of avoidance learning was assessed with three multiple-choice questions, and (c) knowledge about clinical psychology was assessed with four multiple-choice questions and one open-format question(e.g., the open-format question asked participants to write down everything they knew about schizophrenia). For the multiple-choice items, we measured the percentage of correctly answered items per section. To analyze the answers to the open-format questions, we counted the correct statements based on model solutions and also counted the number of technical terms used.

##### Case studies

In a more indirect way, and similar to studies in the domain of medicine (Boshuizen and Schmidt, 1992), we measured clinical competencies (correctness of and explanation supporting diagnosis), and use of higher-order concepts by means of two short case studies: in the first case study, a patient with social phobia was described and in the second case study, a patient with obsessive-compulsive disorder was presented. The description of the first case read as follows (the original material was presented in German):

*Up to now, I was more or less able to avoid unpleasant situations, in my personal life too, by simply leaving when I couldn’t bear it. I seldom attended lectures or courses; nevertheless I managed my studies with acceptable grades. Everybody who does not regularly give presentations is nervous before presenting but with me this is really extreme and I feel physically ill. But that is not my main problem. My sweating makes me sick. I do not sweat in comfortable situations, such as when I am alone at home or with my best friends. But if there is just one stranger with us it starts immediately. My hands turn blue and wet; my shirt gets soaked in cold, clammy and malodorous sweat. I have already consulted a medical practitioner; there are no physiological causes for this. For professional reasons this must stop, I work as a representative*.

*Outsiders see me as a self-confident and competent person; this seems totally absurd to me because I am so nervous that besides the sweating my muscles start shaking (from the legs up to the face). If other people are present, my facial skin turns as pale as ash, my skin gets sweaty, after a while my hair becomes greasy and I feel scruffy even though I am not. Lately, I try to behave confidently, to go up to a person and not to wait till I have to. But afterwards I am so exhausted that I can not stand it any longer and I am exhausted*.

We instructed participants to read the respective case description, recall, and write down important information (recall phase), diagnose, and explain the disorder (explanation phase). In the recall phase, we assessed the amount of correctly recalled statements by comparing recalled statements to presented statements. We assessed the amount of higher-order concepts used by counting the recalled statements that summarized more than one statement presented in the case study. For the diagnosis, a score from 0 (no or wrong diagnosis) to 1.0 (correct and elaborated diagnosis) was assigned. For the explanations we measured the amount of correct statements by comparing them to a model solution compiled by the first author based on textbook knowledge. The model solution included explanations of different positions within psychological therapy, so that in some cases different answers could be counted as correct statement.

##### Interrater-reliability

All answers except the automatically evaluated multiple-choice questions were analyzed by the first author. In order to measure interrater-reliability, 20 randomly selected reactions to the open-format questions and the case studies were independently assessed by a trained research assistant. All intraclass-correlations (ICC, adjusted, single measure) exceeded the criterion of 0.7 (Wirtz and Caspar, 2002).

#### Procedure

Participants individually completed the computer-based questionnaire without access to additional material. They first answered socio-demographic questions and then completed the questionnaire. Multiple-choice questions, open-format questions, and case studies were alternated.

### Results

For all statistical analyses we adopted a significance level of α = 0.05. We conducted a multivariate analysis of variance (MANOVA) with the dependent variables comparing the five groups of participants representing different levels of experience [Pillai’s Trace, *F*(4, 80) = 2.55, *p* < 0.01, η^2^ = 0.36]. The MANOVA shows overall substantial differences between the five groups. Subsequent ANOVAs described below were conducted to test in detail our hypotheses. We computed four *a priori* contrasts, of which only significant or marginally significant contrasts are reported. The first contrast compared novice students to intermediate students, the second contrast compared intermediate students to advanced students, the third contrast compared advanced students to trainee therapists, and the fourth contrast compared trainee therapists to experienced therapists. Cohen’s d is reported as a measure of effect size for the contrast analyses.

There was no significant difference regarding the overall time participants of the five groups spent completing the assessment [*F*(4, 80) = 1.94, *p* = 0.11, η^2^ = 0.09].

#### Knowledge test

The knowledge test applies to all three hypotheses regarding basic psychological knowledge, knowledge of how to apply basics to clinical psychology, and clinical knowledge. Table 1 presents means and standard deviations for the knowledge test.

Regarding basic psychological knowledge, the ANOVA approached significance for the multiple-choice questions [*F*(4, 80) = 2.3, *p* = 0.06, η^2^ = 0.10]. With regard to the open-format question, the ANOVA on the number of correct statements was not significant [*F*(4, 80) = 2.0,*p* = 0.10, η^2^ = 0.09] but the ANOVA on the number of technical terms used revealed a significant effect [*F*(4, 80) = 3.0, *p* = 0.02, η^2^ = 0.13]. Supporting Hypothesis 1 novice students scored the highest in all three measurements. In line with Hypothesis 3 experienced therapists scored rather low on the multiple-choice questions and the amount of correct statements in the open-format question. But it has to be noted that none of the four *a priori* contrasts reached significance.

Concerning the knowledge of how to apply basic psychological knowledge to clinical psychology, the ANOVA revealed significant differences between the groups [*F*(4, 80) = 4.42, *p* < 0.01, η^2^ = 0.18]. As assumed in Hypothesis 1, novice students scored significantly lower than intermediate students [*t*(4, 80) = 3.54, *p* < 0.01, *d* = 1.01]. There was no significant difference regarding the applied knowledge from intermediate students up to the therapists (in line with Hypothesis 2 and 3).

**Figure 1:**
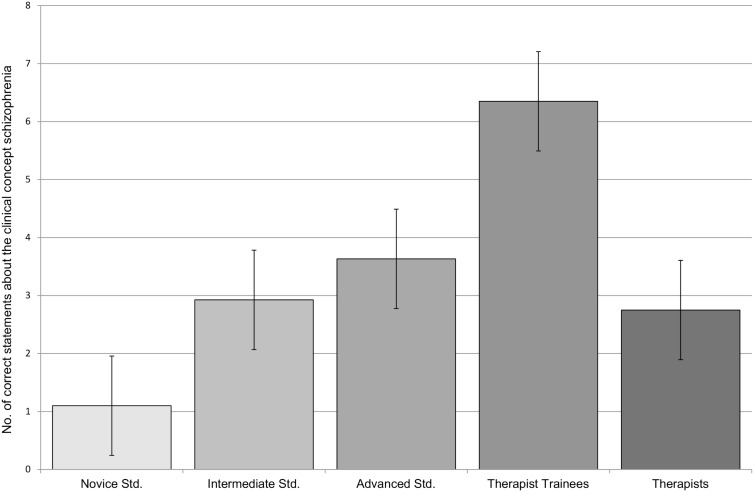
**Results (number of correct statements) for the open-format question regarding the clinical concept schizophrenia**.

In terms of clinical knowledge, the five groups differed significantly on the multiple-choice questions [*F*(4, 80) = 7.7, *p* < 0.01, η^2^ = 0.28], with trainee therapists outperforming even the advanced students [*t*(4, 80) = 2.67, *p* < 0.01, *d* = 0.99]. This result is in accordance with Hypotheses 1 and 2. With regard to the open-format question, the groups likewise differed significantly regarding both the amount of correct statements [*F*(4, 80) = 10.4, *p* < 0.01, η^2^ = 0.34] and the application of technical terms [*F*(4, 80) = 8.3, *p* < 0.01, η^2^ = 0.29]. Figure 1 illustrates this pattern for the number of correct statements. Novice students scored significantly lower than intermediate students [correct statements: *t*(4, 80) = 2.8, *p* < 0.01, *d* = 0.89]; technical terms: *t*(4, 80) = 2.5, *p* = 0.02, *d* = 0.78. This result is in line with Hypothesis 1. Consistent with Hypothesis 2, regarding correct statements, the difference between advanced students and trainees was also significant [*t*(4, 80) = 2.3, *p* = 0.03, *d* = 0.85]. However, contrary to our assumptions in Hypothesis 3, on both variables, experienced therapists scored lower than trainees [correct statements: *t*(4, 80) = 3.8, *p* < 0.01, *d* = *−*1.39]; technical terms: *t*(4, 80) = 3.4, *p* < 0.01, *d* = −1.23.

In summary the results of the knowledge test, reveal that up to the level of trainee therapists, the data reflect what prospective psychotherapists learn in the course of their psychology education and are to a large extent in line with our Hypotheses 1 and 2: novice students performed very well on basic principles of psychology, whereas intermediate students had already learned to apply the basics to clinical psychology, clinical knowledge increased during university training, and further during therapist training. The results in relation to the experienced therapists contradict our Hypothesis 3. Surprisingly, experienced therapists scored significantly lower than trainee therapists regarding clinical psychological knowledge. Unsurprising there was also some evidence that this group had relatively less basic psychological knowledge.

With the two case studies we tested developments in clinical competencies (diagnosis, explanation, use of higher-order concepts in recall) as addressed in all three hypotheses (increase during university training, further increase during therapist training, and even further increase at the therapist level).

#### Case Study 1: social phobia

Means and standard deviations for the dependent variables of Case Study 1 are depicted in Table 2: in the recall phase, the groups differed significantly in the amount of applied higher-order concepts [*F*(4, 80) = 4.8, *p* < 0.01, η^2^ = 0.19] and the amount of recalled statements [*F*(4, 80) = 5.6, *p* < 0.01, η^2^ = 0.22]. Regarding the use of higher-order concepts, as expected in Hypothesis 1 and 2, there was an increase up to the level of trainee therapists but contrary to Hypothesis 3 no increase (but rather some decrease) at the level of experienced therapists. Participants recalled about the same amount of statements from the level of novice up to the trainee therapists, while the experienced therapists recalled fewer statements than the trainee therapists [trainees > therapists: *t*(4, 80) = 3.4, *p* < 0.01, *d* = 1.39].

In terms of the quality of the diagnosis, one of the measures of clinical competence, the groups differed significantly [*F*(4, 80) = 6.7, *p* < 0.01, η^2^ = 0.25]. Concerning Hypotheses 1 and 2, diagnosis improved up to the level of intermediate students but then leveled off [novices < intermediates: *t*(4, 80) = 3.5, *p* < 0.01. *d* = 1.09]. There was no evidence for Hypothesis 3, increase in diagnostic competencies, at the level of experienced therapists.

With regard to explanation of diagnosis, a second measure of clinical competence, the groups differed significantly in the amount of correct statements [*F*(4, 80) = 4.1, *p* < 0.01, η^2^ = 0.17]. Again, the already familiar picture was found, with an increase in correct statements up to the trainees’ level [novices vs. intermediates: *t*(4, 80) = 2.1, *p* = 0.04], *d* = 0.67 but no increase at the experienced therapists level.

Overall in Case Study 1, from novice students up to the trainee therapists, the mean number of correctly recalled statements, mean number of higher-order concepts used, and correctness and explanation of diagnosis increased steadily or leveled off. These results are in line with Hypotheses1 and 2. However, contrary to our expectations in Hypothesis 3 on all variables, the experienced therapists showed no increase, but rather a decrease.

#### Case Study 2: obsessive-compulsive disorder

Means and standard deviations for Case Study 2 are depicted in Table 3. The groups did not differ significantly in the amount of recalled statements [*F*(4, 80) = 1.5, *p* = 0.22, η^2^ = 0.07] but as expected there was a difference in the amount of higher-order concepts used [*F*(4, 80) = 3.1, *p* = 0.02, η^2^ = 0.13]. At the level of advanced students, the number of higher-order concepts significantly increased [intermediates vs. advanced: *t*(4, 80) = 2.4, *p* = 0.02, *d* = 0.59], leveled off at the level of trainee therapists, and showed no further increase at the level of experienced therapists.

In terms of the quality of the diagnosis, the groups differed significantly [*F*(4, 80) = 3.2, *p* = 0.02, η^2^ = 0.14]. In accordance with Hypothesis 1 diagnosis improved at the level of intermediate students [novices vs. intermediates: *t*(4, 80) = 2.2, *p* = 0.03, *d* = 0.64] but then leveled off which was not consistent with our hypotheses.

With regard to explanations, the groups differed significantly in relation to the amount of correct statements [*F*(4, 80) = 5.3, *p* < 0.01, η^2^ = 0.20] which was again mainly due to the difference between novices and intermediates: *t*(4, 80) = 3.5, *p* < 0.01; *d* = 1.09). Overall the data showed an increase up to the trainees (Hypothesis 1 and 2) and then no further increase for the experienced therapists, contrary to Hypothesis 3.

In general, for three of the four variables measured in Case Study 2, we see an increase from novice students to trainee therapists but no further increase but rather some evidence for a slight decrease at the level of experienced therapists. Only regarding the correctness of diagnosis did experienced therapists score similarly to the trainees.

### Discussion of Study 1

The main goal of this study was to shed light on the knowledge of psychologists at different levels of experience in clinical psychology. Students at different stages of their course of studies and experienced psychological therapists completed an instrument assessing basic theoretical and clinical knowledge. Up to and including the level of trainee therapists, our results perfectly mirror the psychology university curriculum in Germany: at the beginning students are mainly taught basic principles of psychology and their applications. Accordingly, we found that students in the beginning stages of their schooling outperformed the other groups on knowledge regarding basic principles. Later on, students also learn clinical knowledge, and we correspondingly found improvements in clinical knowledge at this level. Having graduated from the university, trainee therapists further deepen their knowledge in clinical psychology; and consequently, we found another substantial increase in clinical knowledge and competencies. However, at the level of experienced therapists, our results point to a decrease in knowledge of basic principles and also clinical knowledge and competencies. It should be noted that these therapists did not undergo a full, formalized training comparable to that of today’s trainees. Thus our results could be partly explained by differences among the investigated cohorts. However, the size of the knowledge decrease was remarkable and problematic assuming that knowledge is actually related to the quality of professional action. Our results are not in line with findings in the medical domain that have shown a continuous increase of biomedical knowledge (e.g., Boshuizen and Schmidt, 1992). A possible explanation for this discrepancy may be that our experienced participants had at least 10 years of practical experience, i.e., had graduated from the university many years ago, whereas the experienced participants in the medical studies had a maximum of 4 years of practical experience. And regarding the variables that best resembled practical clinical competencies – the diagnoses – experienced therapists performed as well as trainee therapists.

In Study 1, we assessed retrievable, mostly declarative knowledge. However, expertise in the domain of clinical psychology also includes the ability to deal with complex and dynamically evolving information. Consequently, we examined such competencies in Study 2.

## Study 2

In Study 2, we examined how psychologists at different levels of practical experience process complex and dynamically changing information. We descriptively contrasted the processes of the different groups and evaluated the quality of the outcome. Study 2 complements the quantitative results of Study 1 regarding Hypothesis 2 (increase in clinical knowledge and competencies at the level of trainee therapists), and 3 (increase in clinical knowledge and competencies at the level of experienced therapists) with qualitatively rich data on realistic and complex materials.

### Materials and methods

#### Participants and design

Representing three different levels of clinical experience a total of 15 individuals were interviewed: five advanced university students (mean age in years 25, 60% females) who had already completed their clinical curriculum, five trainee therapists (mean age 30, 40% females) who were at least in their second year of on-the-job training after graduation, and five psychological psychotherapists (mean age 45, 60% females) who had worked for at least 10 years as behavior therapists. None of them had participated in Study 1. All participants received a small financial compensation for their voluntary participation. University students and trainee psychotherapists were recruited during lectures and workshops. The psychotherapists were found *via* a special search engine for physicians and therapists (see text foot note 1) and contacted by phone by the first author.

#### Case-based interview

A case study was realized by means of an interview. Information on the case was presented in writing and consisted of three consecutively presented parts: (1) general information about the patient, (2) details of the case history, and (3) steps in the therapy process the patient underwent. Thus, we were able to evaluate how participants processed and reflected sequentially presented information.

#### Procedure

After informing participants about the interview and audiotaping procedure, we began with the general information about the patient. Participants were instructed to think aloud while working on the case study. After each part, they answered orally presented standardized questions about diagnosis, differential diagnosis, and treatment plan. Altogether, the case-based interview lasted on average 45 min. Finally, participants answered a short questionnaire on socio-demographic data. All participants were interviewed by the first author.

#### Material

In the general patient information, the client was briefly described from the perspective of a general practitioner. The description was focused in particular on the symptoms of a major depression and a herniated vertebral disk. The patient was described to have attempted suicide. He often changed his general practitioner. His personal situation and his family were described briefly as “generally positive.”

Regarding the case history, various life domains were described in more detail: childhood, family, work, attempted suicide, the herniated vertebral disk, and leisure time. All of these domains were characterized by unstable interpersonal relationships and employments. According to the description, the patient behaved in a self-destructive and highly risky way, and smoked heavily.

With regard to the therapy process, a female therapist was introduced who diagnosed a major depressive disorder and started cognitive-behavioral therapy. In the beginning, the patient seemed to be highly motivated but then problems arose that mainly resulted from his unstable and impulsive behavior. The therapist created an inappropriate therapist-client relationship. The patient was described to show symptoms of borderline personality disorder, e.g., unstable interpersonal relationships, unstable affect, impulsivity, and an unstable self-image. Finally, it was said that the patient lost his job, abandoned therapy, and was suicidal. The full text of the case study is available in German on http://www.psychologie.uni-freiburg.de/abteilungen/Allgemeine.Psychologie/s2exp.

#### Analyses

Supervised by the third author, a very experienced psychological therapist and supervisor, the fourth author evaluated the transcribed “thinking aloud” protocols blindly, i.e., without knowing the respective participant’s level of experience. The evaluation was based on an exemplary case formulation. The exemplary case formulation consisted of three parts: (1) the correct diagnoses and their explanations: major depressive episode, borderline personality disorder, and addiction to cigarettes, (2) main components of a case conceptualization (see Grawe, 2004): therapy motivation, behavior in interpersonal relationships, resources, critical life events, and disorder-specific components such as emotion regulation (3) an adequate treatment plan, for example as defined by dialectical-behavior therapy, schema therapy, or cognitive-behavioral therapy. Other models of psychological therapy were possible if the therapy steps were accepted state of the art for the treatment of the diagnosed disorders. Based on this exemplary case formulation, the thinking aloud protocols and answers to the questions were evaluated. For each protocol, an overall rating from 1.0 (lowest value) to 6.0 (highest value) was assigned. Additionally, the fourth author provided a written qualitative evaluation.

### Results

#### Overall ratings of the quality of the participants’ information processing of the case study

The mean of the overall ratings of the students was 4.55 (SD = 0.57); the trainees achieved a mean of 5.4 (SD = 0.38), and the experienced therapists reached a mean of 4.85 (SD = 0.68). Apart from one positive outlier with a value of 5.5, the five students achieved only values of 4.0, 4.25, 4.5, and 4.5. Similarly, the trainees’ values were rather homogeneous: 5.0, 5.25, 5.25, 5.5, and 6.0. The results of the experienced therapists demonstrated more variance with values of 4.0, 4.5, 4.75, 5.25, and 5.75. These results are in line with Hypothesis 2 (increase for the trainees) but not with Hypothesis 3 (increase for the experienced therapists).

The next paragraphs contrast the case-based statements we found at the different experience levels on (1) diagnostic statements concerning the more difficult diagnosis of borderline personality disorder, (2) the case conceptualizations, and (3) the proposed treatment.

#### Diagnosis borderline disorder

Generally, advanced students were not able to diagnose the borderline personality disorder. Indeed, three of them identified the relevant symptoms but failed to integrate them. All trainee therapists recognized the symptoms and integrated them into the diagnosis of a personality disorder. However, three of them neglected important information, like for example the unstable relationships and inferred a wrong but related diagnosis, e.g., narcissistic personality disorder. The experienced therapists diagnosed very heterogeneously. On the one hand, three of them mentioned borderline personality disorder, yet three of them still proposed the wrong diagnosis. These results concerning the quality of the diagnosis are in line with Hypothesis 2 but not with Hypothesis 3.

To illustrate these differences between the three levels of experience we provide exemplary statements from participants of the three different groups translated from German to English.

(a) Subject 6, a student, diagnosed wrongly:*[*…*] now family situation, ok, so there seem to be problems after all, so he’s often checking, whether he’s saying the truth, well maybe he somehow has like obsessive thoughts, compulsive checking or hallucinations [*…*]*
(b) Subject 9, a the trainee therapist, justified his correct diagnosis with symptoms described in the case study: *[*…*] for the borderline- diagnosis, what do we have here? We have the inner emptiness, we have suicidality, we have the self-harming behavior, at least with extreme sports, we have the suicidality, also this fear of abandonment, impulsive behavior. Of course we also have, that he even in his self-image, could be such an indication for such an unstable self-image, too, in that he suddenly thinks, they all would consider him to be antisocial and things like that, and also these breakdowns [*…*]*
(c) Subject 4, an experienced therapist, diagnosed wrongly due to deficient clinical knowledge: *[*…*] that is this narcissistic disorder, I would pin that on him additionally, because it emerges from this presumed mother-relationship. From experience all sons, who with a single superior mother, who maybe also have depressive anxious traits, in fact, have in the background such a narcissistic disorder [investigator: asks for explanatory statement] the anger. [*…*]*

#### Case conceptualizations

We checked the statements of the participants of our study for the main components of a case conceptualization (see Grawe, 2004): therapy motivation, behavior in interpersonal relationships, resources, critical life events, and disorder-specific components such as emotion regulation. Four out of five advanced students recognized therapy motivation as an important factor; however, two of them were not aware of the problematic aspects of the seemingly motivated behavior of the patient at the beginning of the therapy. This highly emphasized therapy motivation was problematic since it contributed to unrealistic expectations of the patient. Advanced students often mentioned the patient’s unstable relationships and problems with emotion regulation, but they failed to integrate them into a coherent case conceptualization. Resources and critical life events were reflected.

Trainee therapists were surprised by the highly motivated behavior of the patient at the beginning. Three of them expected that patients’ motivation would eventually change to refusing therapy All trainee therapists adequately reflected the patient’s interpersonal relationships and they also were aware of his impulsivity. Resources and critical life events were also reflected. The trainees integrated the information that was provided into a meaningful individual case conceptualization. Based on this deep understanding, they sometimes predicted some of the problems that indeed would arise in a later phase.

Therapists were not surprised by the patient’s high motivation for therapy at the beginning; two of them were able to reflect on and offer an interpretation of therapy motivation, for example as an attempt to impress the therapist. However, most therapists did not recognize the problematic aspect of this seemingly high therapy motivation that contributed to unrealistic expectations of the patient. This heterogeneous picture also emerged regarding interpersonal relationships, emotion regulation, and impulsivity of the patient: some therapists reflected them very conscientiously, others only superficially, or without reference to the described case. These results on the quality of the case conceptualizations are again in line with Hypothesis 2 but not with Hypothesis 3. Again we provide exemplary statements from participants of the three different groups.

(a) Subject 11, a student, showed difficulties regarding the integration of information: *[*…*] then there increasingly are tantrums, especially, when he feels criticized, which I find consistent with the hypothesis now in a sense, because I would have assumed, at work, too, situations of criticism or situations of social degradation trigger a reaction in him, which are cause for the colleagues’ bullying [*…*]*
(b) Subject 9, a trainee therapist, predicted problems that arose later: *[*…*] so one has to look now, whether out of this somehow a life pattern has somehow formed, that somehow in relationships again and again losses happen to him or that he leaves people or is unstable in relationships, but no idea, I have to look [*…*]*
(c) Subject 2, an experienced therapist, focused on a relevant factor, interpersonal relationships but reflected it on a superficial level: *[*…*] tense, all these relationships are somehow tense [*…*]*

#### Treatment plan

Three of the advanced students described therapeutic approaches that would likely lead to a termination of therapy such as choosing a confrontational approach. One of the advanced students described an adequate treatment plan; however, it remained on a rather general level. Although some trainee therapists showed deficits regarding the correct diagnosis (see above), all of them described adequate treatment plans, e.g., as defined by dialectical-behavior therapy for borderline personality disorders. They emphasized the unconditional positive regard and the need to proceed cautiously and slowly. With experienced therapists we again obtained a mixed picture. About half of the therapists described adequate therapeutic approaches and also considered the therapeutic relationship. The other half described rather general approaches. These results on the quality of the treatment plans again are in line with Hypothesis 2 but not with Hypothesis 3. Four exemplary statements are provided for illustrative purposes.

(a) Subject 11, a student, proposed an unsatisfactory treatment plan although the complete case study was already presented: *[*…*] I would explicitly address my role, meaning that I am not prepared, to collude with him, but rather, that I expect, that he works on himself, because otherwise it would again go in the direction of the therapy having to fail, precisely because he shifts the blame on others and doesn’t look for it [the blame] in himself, like in the current example, has been the case with the layoff as well [*…*]*
(b) Subject 7, a trainee therapist, proposed a satisfactory treatment: *[*…*] I’d be careful in any case, I would very much keep it in the back of my mind, that in any case the relationship is very important and I would pay a lot of attention to his not having the feeling to be seen as inferior, deficient, but rather really look, at what kind of resources, options he does have [*…*]*
(c) Subject 6, an experienced therapist also proposed a satisfactory treatment: *[*…*] this thing with the two-timing would be the critical thing, so like dialectical so to speak, that one says, that is totally okay, how he is, that is legitimate, one can infer that, how he is, and although it is okay how he is, it would probably nevertheless also be sensible, to change, and that that I think, that is just important, that one takes that along from the get-go, so to take him seriously on the one hand in his in his so-being, yeah so whatever there is now and to question that, one can do that, when the relationship has grown, the therapeutic relationship thus has to be resilient [*…*]*
(d) Subject 2, also an experienced therapist, would refer the patient to another therapist: *[*…*] he’d have to go to a man, I’d first of all say that [*…*] I don’t think, that a therapist can do anything now, that could lead to, with this patient, as it were, that he sticks with his problems, because the problem in his precisely in his case is difficult [*…*] I wouldn’t know at the moment, how I could solve that, I have no idea to this end, I’d have to go to supervision, or ask my colleagues [*…*]*

### Discussion of Study 2

In Study 2, we found distinct differences between the three examined levels of clinical experience. The groups differed regarding the quality of diagnosis, case conceptualizations, as well as planning of the treatment. Consistent with Hypothesis 2, advanced university students had more difficulties in dealing with the provided complex clinical case study compared to the trainee therapists, but they were able to diagnose the major depression and to plan adequate general treatment for it. They failed in relation to making a sufficiently sophisticated and complex diagnosis and treatment plan for borderline personality disorder and addiction. Regarding the experienced psychotherapist, we obtained mixed results that are not in line with Hypothesis 3; some of them worked on the case study in an almost exemplary manner; they integrated the provided information and supported their diagnoses with symptoms described in the case study. However, other experienced therapists failed to develop an individual case conceptualization, did not diagnose correctly, or failed to plan a treatment that was based on the information described in the case study. As in our first study and consistent with Hypothesis 2, the trainee therapists achieved the best results. They integrated the described symptoms into adequate diagnoses, developed meaningful case conceptualizations, and planned satisfactory treatments.

## Overall Discussion and Conclusion

We assessed the effects of university training, postgraduate psychotherapist training, and clinical experience on expertise in clinical psychology. A central characteristic of our approach was to ensure that the expertise in our research group comprised competencies in the assessment of cognitive processes as well as expertise in clinical psychology and psychotherapy. Thus, our research group consisted of both cognitive scientists and researchers from clinical psychology. This collaboration enabled the construction of relevant materials that partly bridged the gap between directly assessing knowledge about facts and concepts and indirectly assessing procedural knowledge for solving complex and dynamically changing clinical cases. We consider this multi-method approach to be a clear advantage in expertise research in domains like clinical psychology.

Knowledge about facts, concepts, and procedures has been shown to be one of the main factors discerning experts and novices in different fields (e.g., Bédard and Chi, 1992). Thus, our first study aimed at assessing these types of knowledge at different levels of experience, i.e., in different stages of studying psychology, when being trained as a psychotherapist, and when having worked for years as a psychotherapist. A strong knowledge base is a prerequisite for clinical expertise; however, psychotherapy also includes the ability to deal with complex and dynamically evolving information. We examined these latter aspects in a second study by means of a case-based interview. Taking this approach, we tried to complement a more traditional way of analyzing knowledge with a form of assessment that simulates the high demands psychotherapists face in their work. While expertise research often indirectly deduces the available knowledge, in our research we additionally employed direct measurements of knowledge.

In summary, our studies revealed that expertise development up to and including the level of trainee therapists perfectly mirrors the organization of the university curriculum in psychology and therapist training in Germany. This demonstrates the positive effects of academic studies and subsequent professional training on expertise development. However, at the level of experienced therapists, the obtained picture is not so bright. Our results point to a decrease in knowledge and variability in clinical competencies. In the following paragraphs, we discuss the results of the two studies with respect to our research questions (cf. Introduction) in more detail.

Effects of studying psychology at the university on expertise (Hypothesis 1): in the first 3 years of studying psychology, university students learn about basic concepts and principles of psychology and their application. Consequently, students at these levels outperformed participants of the other levels on these variables. Later on in their course of studies, students who chose clinical psychology as their main focus showed clear improvements in clinical knowledge. They also were able to provide correct diagnoses in short case studies. However, the case-based interview revealed that they failed to integrate information in a complex and dynamically changing situation leading to incomplete diagnoses and inadequate treatment planning.

Effects of postgraduate therapist training on expertise (Hypothesis 2): since 1999, after attaining the Master’s or Diploma degree, future psychotherapists must engage in a certified postgraduate training program comprising theoretical clinical courses, practical training, and self experience. In both of our studies, trainee therapists achieved the best results on nearly all measured variables. In particular, our results indicate a further increase in clinical knowledge and competencies. Trainee therapists provided high quality diagnoses, coherent case conceptualizations, and adequate treatment planning. We conclude that the training program is very successful in substantially enhancing clinical competencies.

Effects of more than 10 years of practical experience on expertise (Hypothesis 3): the experienced therapists who participated in our studies had been working for at least 10 years in a clinical setting. They had not engaged in the above described therapist training but they had also completed their university studies with a “Diploma” degree and had completed a further but less standardized therapist training. At the level of these experienced therapists, our results point to a decrease both in basic psychological knowledge and knowledge of how to apply basics to clinical. Surprisingly, we observed even some decrease in clinical knowledge in contrast to Hypothesis 3, which stated that experienced therapists show more clinical knowledge than the less experienced groups. The decrease of clinical knowledge at the level of experienced therapists seems to contradict findings in other domains, in particular medicine (e.g., Boshuizen and Schmidt, 1992). An explanation for this contradictory result may be that experienced participants in our studies had completed their university studies at least 10 years ago, whereas the experienced participants in the medical studies had graduated only a few years ago. The experienced psychotherapists generally provided correct diagnoses in the short and simple structured case studies of Study 1. However, regarding the case-based interviews in Study 2 a mixed picture emerged for the experienced therapists: on the one hand, two of the five experienced therapists worked on the case study in an almost ideal way; on the other hand, three experienced therapists did not develop an individual case conceptualization, their diagnoses were incomplete or wrong, and they did not adapt their treatment plan to the patient’s history. This finding is in line with studies showing large variance in therapy outcomes across therapists (e.g., Luborsky et al., 1997; Beutler et al., 2004).

Several limitations of our empirical investigations should be noted: (a) both studies were cross-sectional studies, examining different cohorts. Our sample of experienced therapists started working as psychotherapists before the psychotherapists’ law came into effect in 1999. Thus, compared to the sample of trainee therapists, the experienced therapists had completed a less stringent therapist training after completing their university studies. (b) University studies in psychology are now organized such as to lead to a Bachelor’s and a Master’s degree. The participants in our studies still graduated with a diploma degree. But as the Master’s and the “Diploma” degrees are comparable in content, methods, and process, it seems safe to assume that the obtained results would be similar with the new sequence of degrees. (c) The studies were conducted in a non-psychotherapeutic setting. Thus, the findings cannot be generalized directly to the behavior in such a setting. (d) The approach of case-based interviews is very time-consuming regarding analysis and evaluation of data. However, further studies are needed to replicate our results with a bigger sample size. (e) It must also be noted, that personality disorders were not commonly included in many training programs until recent times and clinical knowledge about personality disorders has developed a lot over the past 10 years.

Similar to research in other domains, our studies revealed that in the domain of clinical psychology experience does not automatically translate into expertise (Eells et al., 2005; Davis, 2009). Expertise can only develop if the future expert engages in a substantial amount of deliberate practice (Ericsson et al., 1993). So, how can we ensure that practice and experience is transformed into expertise in the domain of clinical psychology?

Our findings imply that the approach of combining standardized university studies in psychology with a focus on clinical psychology followed by a substantial theory based practical training in psychotherapy is very successful. This training is characterized by linking advanced theoretical clinical courses with clinical practice. Clinical courses held by practitioners with a strong scientific background are taught in a problem-based way (e.g., Schank et al., 1999), thus facilitating memory recall and the application to clinical problems and reducing inert knowledge (e.g., Renkl et al., 1996). Inert knowledge is understood to be theoretical in nature and not applicable to practical problems.

Most surprising are our findings regarding the experienced therapists with more than 10 years of clinical experience. In order to stay licensed, all psychotherapists in Germany have to participate in continuing professional development classes. However, this continuing education seems to be successful only in some cases. One explanation may be that practitioners need to be able to identify areas where there is a need for improvement in order to find (and sign up for) appropriate courses. However, in the domain of medicine, it was found that self-assessments barely correlate with outcome criteria (Davis, 2009). The problem of self-assessment also applies to supervisory settings because the supervisee him/herself decides which problem he or she wants to work on.

Our studies reveal that university training and therapist training promote the development of expertise in clinical psychology and psychotherapy. However, the trainee therapists rather than the experienced therapists did best in our studies. We conclude that at least under the given conditions a central component of evidence-based practice in psychology (APA, 2006), namely clinical expertise, is not ensured by several years of clinical experience. Expertise needs ongoing and supervised deliberate practice (Ericsson et al., 1993) and therapists must be strongly supported to meet this challenge.

## Conflict of Interest Statement

The authors declare that the research was conducted in the absence of any commercial or financial relationships that could be construed as a potential conflict of interest.

## Footnotes

^1^Today, students of psychology at the University of Freiburg are enrolling in Bachelor’s and Master’s programs. Our description applies to the diploma curriculum, which has recently been replaced, by the Bachelor’s and Master’s program but content and procedure of both university trainings are virtually the same.

^2^http://www.arztsuche-bw.de/

^3^http://www.psychologie.uni-freiburg.de/abteilungen/Allgemeine.Psychologie/s2exp
